# Trainspotting in a cilium

**DOI:** 10.7554/eLife.32473

**Published:** 2017-10-30

**Authors:** Dhivya Kumar, Stephen M King

**Affiliations:** 1 Department of Biochemistry and BiophysicsUniversity of California, San FranciscoSan FranciscoUnited States; 2 Department of Molecular Biology and BiophysicsUniversity of Connecticut Health CenterFarmingtonUnited States

**Keywords:** intraflagellar transport, kinesin, dynein, ciliary length control, single molecule imaging, photogate, Other

## Abstract

A new imaging technique sheds light on how cilia regulate their length and growth.

**Related research article** Chien A, Shih SM, Bower R, Tritschler D, Porter ME, Yildiz A. 2017. Dynamics of the IFT machinery at the ciliary tip. *eLife* **6**:e28606. doi: 10.7554/eLife.28606

Cilia are antenna-like structures that protrude from many cells and perform a variety of roles: some help cells to move while others are involved in signaling. Faulty or disrupted cilia can lead to diseases known as ciliopathies that can affect multiple organs and cause symptoms ranging from blindness, kidney cysts and neurological problems to infertility and skeletal malformations (Reiter and Leroux, 2017).

Cilia are complex structures that contain over 600 proteins, all of which have to be transported to the cilium when it is being assembled. Moreover, all the proteins and receptors involved in signaling have to be shuttled into and out of the cilia as needed: this is done by an elaborate piece of molecular machinery called the intraflagellar transport system, or IFT for short (Lechtreck, 2015).

This system was first discovered in the single-celled green alga *Chlamydomonas reinhardtii*, whose cilia share many similarities with those found in mammals (Kozminski et al., 1993). In *Chlamydomonas* the components of the IFT system form complexes known as IFT trains, which carry cargo proteins along dedicated microtubule tracks (that run along the length of cilia) in different directions (Figure 1). Kinesin motor proteins move the IFT trains from the base of the cilia to the tip, while dynein motors move the IFT trains back to the base (Lechtreck, 2015).

**Figure 1.**
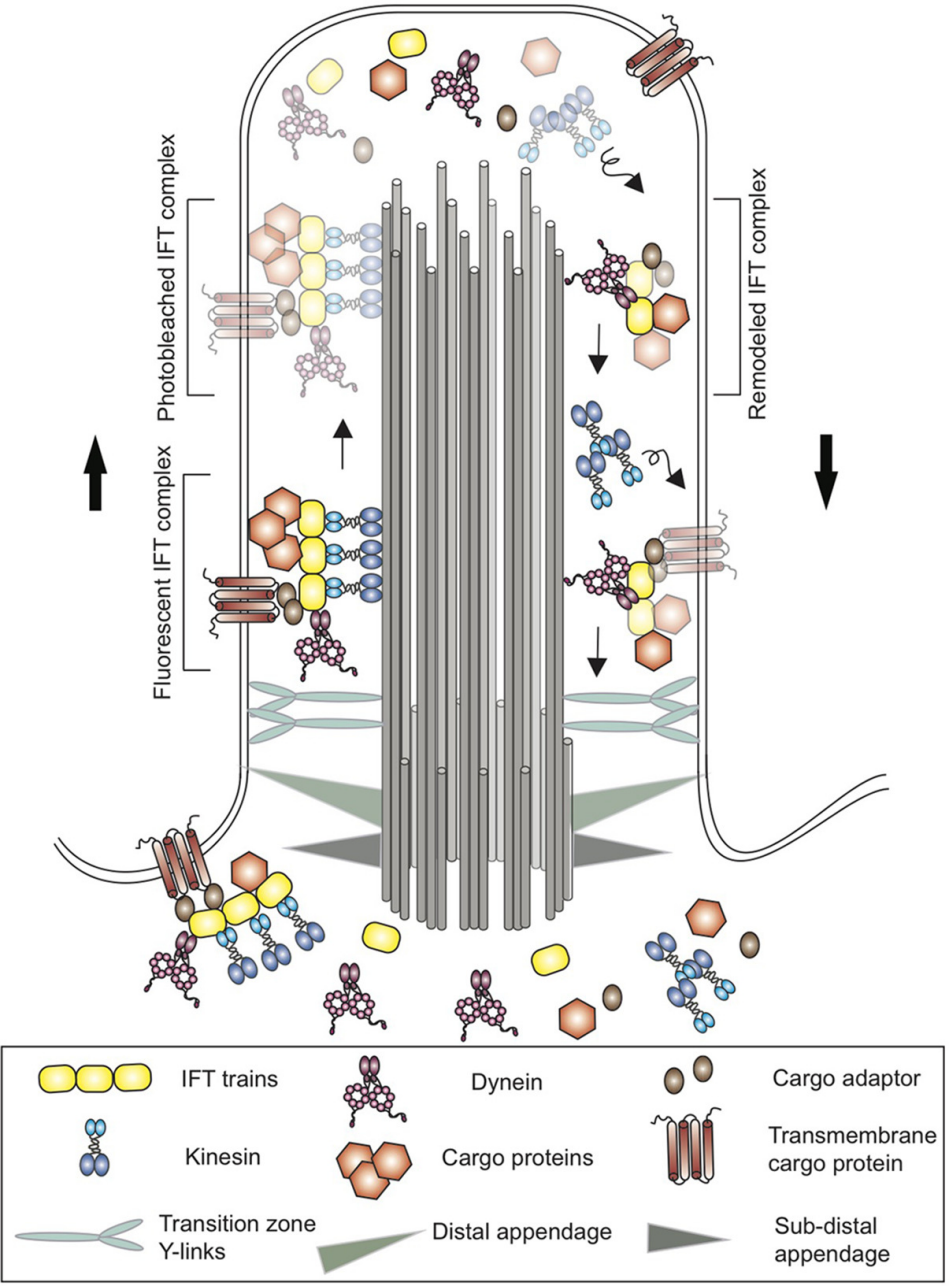
Schematic of a cilium in the green alga *Chlamydomonas*. Cilia are anchored to the cell membrane by distal and sub-distal appendages (grey and green triangles) in the basal body. The Y-links (light blue) in the transition zone gate at the base controls the entry of proteins into the cilium. Proteins (brown hexagons), transmembrane proteins (brown) and other cargo are transported along microtubule tracks (grey cylinders) from the base of the cilium to the tip by kinesin motor proteins (blue) and an IFT train (yellow). The cargo proteins are attached directly to the IFT train or via cargo adaptors (brown ovals). At the tip, the trains release their cargo and break apart before remodeled IFT trains are returned to the base by dynein (pink). Kinesin, on the other hand, diffuses back to the base. Using a new imaging technique called PhotoGate, Chien et al. labeled certain proteins with fluorescent molecules, and then used a laser to 'photobleach' most of them (represented here by fading) before they reached the tip. Since the photobleached molecules are dark, it is possible to follow the small number of labeled trains that remain fluorescent, and to better track their behavior at the tip of the cilium in order to study the remodeling of IFT trains.

The tip of a cilium is a busy place and once the IFT trains have reached the tip and unloaded their cargo, they undergo major remodeling before returning to the base with their new cargo. However, until now existing microscopy techniques have not been able to reveal what happens during this remodeling. Now, in eLife, Ahmet Yildiz at the University of California Berkeley and colleagues – including Alexander Chien and Sheng Min Shih as joint first authors – report new insights into the behavior of IFT trains and motor proteins (Chien et al., 2017).

Chien et al. used a method called PhotoGate microscopy to image the tips of cilia in *Chlamydomonas* and to track individual IFT trains (Belyy et al., 2017). With this technique, most of the IFT complexes labeled with a fluorescent marker were ‘photobleached’ by moving a laser from the tip of the cilium to the base, leaving only a few selected complexes fluorescent. This way, the entire journey of individual trains to the tip of the cilium and back to the base could be tracked. Chien et al. found that the IFT trains stop at the ciliary tip for about three seconds, during which they undergo extensive remodeling. The trains split apart and mix with other ones to form new trains – a process that takes just over a second – and then wait for two seconds before departing. For every fluorescent train arriving at the tip, about 2.4 new fluorescent ones return to the base.

Next, Chien et al. tracked the movement of kinesin-II and dynein-1b and found that although these two motor proteins arrive together at the tip of the cilium, they depart independently of each other. While dynein-1b participates in the formation of new trains, kinesin-II rests at the tip for about two seconds and is not part of the new trains. Rather, kinesin-II seems to rely on passive diffusion rather than active transport to return to the base of the cilium, which means that it takes 10 times longer to return than dynein-1b.

In most eukaryotic cells, the length of cilia is tightly regulated, and if they are amputated, cilia grow back to the same length as before (Ishikawa and Marshall, 2017). But how do cells know how long a given cilium is? The results of Chien et al. suggest that the availability of kinesin-II at the base provides this information: when a cilium is short, it takes a relatively short time for kinesin-II to diffuse back to the base; however, as the length of the cilium increases, it takes longer for kinesin-II to diffuse back, and its availability to power new IFT trains is reduced, as is the growth rate of the cilium.

This study is one step toward a better understanding of the workings of IFT trains and how cilia regulate their length. However, in some species, such as worms and mammals, kinesins rely on the IFT trains rather than diffusion to return to the base, so it remains unclear how cilia length is maintained in these organisms (Mijalkovic et al., 2017; Prevo et al., 2015; Williams et al., 2014). Previous research suggests that several kinases affect the length of cilia, but it is not yet known if these kinases control kinesin motor levels at the base of cilia, or work in a different manner to maintain cilia length (Ishikawa and Marshall, 2017). Mutations affecting dynein have been found in several severe ciliopathies and could also affect the cilium length, which highlights how important length control is for cilia to work properly (McInerney-Leo et al., 2013).
